# Discoloration Potential of Endodontic Sealers: A Brief Review

**DOI:** 10.22037/iej.2016.20

**Published:** 2016

**Authors:** Sohrab Tour Savadkouhi, Mahta Fazlyab

**Affiliations:** a *Department of Endodontics, Dental Branch, Islamic Azad University of Medical Sciences, Tehran, Iran;*; b * Iranian Center for Endodontic Research, Research Institute of Dental Sciences, Dental School, Shahid Beheshti University of Medical Sciences, Tehran, Iran*

**Keywords:** Root Canal Treatment, Root Canal Sealer, Spectrophotometry, Tooth Discoloration

## Abstract

Tooth discoloration induced by endodontic sealers, is a common finding that impairs aesthetic outcome of endodontic treatment. The aim of the present mini literature review, was to summarize the existing data on discoloration potential of different endodontic sealers. The research covered the article published in PubMed and Google Scholar from 2000 to 2015. The searched keywords included ‘tooth discoloration AND endodontic’, ‘tooth discoloration AND sealer, ‘tooth discoloration AND zinc-oxide eugenol sealer’, ‘tooth discoloration AND Calcium Hydroxide Sealer’, ‘tooth discoloration AND Glass Ionomer Sealer’, ‘tooth discoloration AND epoxy-resin Sealer’, ‘tooth discoloration AND Silicon Based Sealer’, ‘tooth discoloration AND Bioceramic Sealer’ and ‘Spectrophotometry’.

**Conclusion:**

A total number of 44 articles were gained which reduced to 11 after excluding the repetitive items. The available evidence for discoloration potential of endodontic sealers currently available on the market is scarce. However, it can be concluded that all endodontic sealers can potentially stain the tooth structure to different degrees.

## Introduction

Sealer-induced tooth discoloration subsequent to endodontic treatment, is a common finding that impairs the aesthetic outcome [1]. This discoloration is consequence of sealer compounds that spread into dentinal tubules during or after its setting [2]. Certain components such as eugenol, phenol and silver additives may be the causes of coronal discoloration [3-5]. Bleaching of iatrogenically discolored teeth is more difficult, time consuming and less effective compared to traumatically discolored teeth [6]. Therefore, in order to select the most appropriate sealer, it is important for dental professionals to have a thorough understanding of their discoloration potential. For assessment of tooth discoloration, many procedures are introduced including dental color matching instruments and systems like spectrophotometric analysis and Photoshop software which are more accurate and more reproducible compared to shade assessment by unarmed human eye [7]. In a comprehensive review by Ahmed *et al*. [8] they concluded, all endodontic sealers cause tooth discoloration when left in pulp chamber. For example AH-26 in a complex environment inside the root canal system triggers a chemical interaction that results in conversion of filler to bismuth compound, which become green to black colors. Corrosion of silver also results grey to black discoloration. Modified AH-Plus contains zirconium oxide as opacifier has long-term color stability. 

Cleaning the pulp chamber after obturation by cotton pellet soaked with absolute alcohol is essential. Presence of sealers in pulp chamber together with a defective or metallic restoration, worsen the condition as combined etiologic factor.

In a review study, Krastle *et al*. [9] showed that all materials used in modern endodontics might stain teeth. For example, silver ions in AH-26 and Pulp Canal Sealer cause severe discoloration of teeth and this action might not be necessarily associated with tubule penetration of these sealers.

The aim of this review was to summarize the existing literature published from 2000 to 2015 about the discoloration potential of sealers used for endodontic procedures.

## Materials and Methods

A comprehensive English-limited search was done in PubMed and Google Scholar on the manuscripts published from 2000 to 2015, using the following keywords: ‘tooth discoloration AND endodontic’, ‘tooth discoloration AND sealer’, ‘tooth discoloration AND zinc-oxide eugenol sealer’, ‘tooth discoloration AND calcium hydroxide sealer’, ‘tooth discoloration AND glass ionomer sealer’, ‘tooth discoloration AND epoxy-resin Sealer’, ‘tooth discoloration AND silicon based sealer’, ‘tooth discoloration AND bioceramic sealer’ and ‘spectrophotometry’. Then, a hand-search was done in the references of collected articles to find more matching papers.

## Results

A total of 44 articles were found which in order of their related keywords are ‘tooth discoloration AND endodontic’ (1 article), ‘tooth discoloration AND sealer (16 articles)’, ‘spectrophotometry AND endodontic’, ‘tooth discoloration AND zinc-oxide eugenol sealer (6 articles)’, ‘tooth discoloration AND calcium hydroxide sealer (4 articles)’, ‘tooth discoloration AND glass ionomer sealer (0 articles)’, ‘tooth discoloration AND epoxy-resin Sealer (9 articles)’, ‘tooth discoloration AND silicon based sealer (0 articles)’ and ‘tooth discoloration AND bioceramic sealer (1 articles)’. After checking the titles and excluding the repetitive titles, 11 articles remained (Table 1). The study techniques included spectrophotometry (5 studies) and comparative before-and-after images (5 studies).

***Zinc oxide eugenol base sealers***

Zinc oxide-eugenol (ZOE) sealers have a history of successful use over an extended period of time. An advantage of these types of sealers is their profound antimicrobial activity [10]. However the main disadvantage is its resorption after extrusion into the periradicular tissues [11]. Sealers like Rickert, Pulp Canal Sealer (SybronEndo, Orange, CA, USA) and Pulp Canal Sealer EWT (extended working time), Roth’s Sealer (Roth International, Chicago, IL, USA), TubliSeal (SybronEndo, Orange, CA, USA) and Wach’s Sealer (Balas Dental, Chicago, Illinois) exhibit a slow setting time (which is compensated in EWT version of TubliSeal and Pulp Canal Sealer), shrinkage on setting, solubility and tooth discoloration [9, 12-14]. 

In an *in vitro* spectrophotometric analysis of crown discoloration by loannidis [15] it was shown that Roth 811 induced severe coronal discoloration in comparison with MTA Fillapex. Meincke *et al.* [16] also used the similar technique to compare the discoloration potential of Sealer 26 (epoxy-based resin; (Dentsply, Petropolis, RJ, Brazil), Endomethazone (a medicated sealer with formaldehyde; Specialites, Septodont, Saint-Maur, France), AH-Plus (epoxy resin sealer; Dentsply, Tulsa Dental, Tulsa, OK, USA) and Endofill (a ZOE-based sealer; Herpo Produtos Dentários Ltda, Petrópolis, RJ, Brazil) and reported higher tooth discoloration with the former two sealers. Another *in vitro* study compared the pre- and four-month post treatment photographs of treated teeth and confirmed the minimal crown discoloration induced by Dorifill in comparison with AH-26 sealer and stated that ZOE-based sealers may be more appropriate for root canal treatment of anterior teeth [17]. Davis *et al.* [4] have also examined the coronal discoloration potential of Sealapex, Roth 801 (Kerr, Romulus, MI., USA) and AH-26 (Detrey, Dentsply, Germany). They stated that all sealers cause different degrees of coronal discoloration irrespective of their type which occurred within few weeks; however, the greatest amount of discoloration was observed with AH-26.

In a brief view it can be concluded that ZOE-based sealers have low discoloration potential and can be considered more appropriate for endodontic treatment in esthetic zones.

***Calcium hydroxide-based sealers***

Calcium hydroxide sealers were developed and admired for their antimicrobial activity. It was thought that these sealers have some osteogenic-cementogenic potential, as well [18]. Unfortunately, these actions have not been proved yet. Solubility of the sealer is a perquisite for release of its calcium hydroxide content and sustained activity [18, 19]. This mechanism of action is however inconsistent with the purpose of a sealer [19]. Some of the manufactured commercially available calcium hydroxide-based sealers are Calciobiotic Root Canal Sealer (CRCS, Hygienic, Akron, OH, USA), Sealapex (SybronEndo Corporation, Orange, CA, USA), Apexit and Apexit Plus (Ivoclar Vivadent, AG, Schaan, Liechtenstein). 

In an *in vitro* study using digital imaging technique, Parsons *et al.* [2] compared the discoloration potential of AH-26, Kerr Pulp Canal Sealer, Roth 801 and Sealapex and reported slightly more discoloration with AH-26 and Kerr Pulp Canal Sealer. They concluded that almost all endodontic sealers cause slight to moderate and generally progressive discoloration over 12 months [2]. Davis *et al.* [4] conducted an *in vitro* study by digital imaging technique to evaluate the tooth discoloration and amount of sealer penetration into dentine after using AH-26, Kerr Pulp Canal Sealer, Roth 801 and Sealapex. They reported no measurable penetration of sealer into dentin for all groups and no dentin discoloration. However, notable discoloration occurred in the sealer bulk and after two years the discoloration remained confined primarily to the pulp chamber [4].

**Table 1.** Included studies on tooth discoloration induced by root canal sealers

**Author**	**Test material(s)**	**Study type**	**Method**	**Sample size**	**Result**
**Ioannidis ** ***et al.*** ** [** 15 **]**	MTA Fillapex Roth 811	*Ex vivo*	UV-VIS spectrophotometer	45	MTA Fillapex resulted in minimal discoloration, while Roth 811 induced severe discoloration
**Jahromi ** ***et al.*** ** [** 17 **]**	AH-26 Dorifill (ZOE)	*Ex vivo*	Digital images and photoshop	50	AH26 had greater discoloration (*P*<0.05)
**Davis ** ***et al.*** ** [** 4 **]**	AH-26 Kerr Pulp Canal Sealer Roth 801 Sealapex	*Ex vivo*	Digital images	50	There was no measurable penetration of sealer into dentin for all groups and no dentin discoloration occurred
**Meincke ** ***et al.*** ** [** 16 **]**	AH-Plus Endofill Endométhasone Sealer 26	*Ex vivo*	Spectrophotometer	40	Sealer 26 and Endomethasone producing the greatest discoloration
**Parsons ** ***et al.*** ** [** 2 **]**	AH-26 Kerr Pulp Canal Sealer Roths 801(nonstaining) Sealapex	*Ex vivo*	Digital images	-	Slightly more discoloration with AH-26 and Kerr Pulp Canal Sealer observed
**El Sayed and Etemadi [** 20 **]**	AH-Plus Apexit Plus Sultan Amalgam Distilled water	*Ex vivo*	Shadepilot and spectrophotometer	50	Apexit plus showed the lowest coronal discoloration effect compared to other sealers
**Lenherr ** ***et al.*** ** [** 21 **]**	Blood calcium hydroxide ApexCal Ultracal XS Ledermix triple antibiotic paste (3Mix) Grey MTA (GMTA), GMTA + blood White MTA (WMTA) WMTA + blood, Portland cement (PC) PC + blood AH-Plus.	*Ex vivo*	Standardized colour measurement (VITA Easyshade compact)	210	Most discoloration was measured in 3Mix and Ledermix The lowest colour change values were observed in AH-Plus, PC, calcium hydroxide, Ultracal XS, and WMTA (*P*<0.0001)
**Partovi ** ***et al. *** **[** 5 **]**	AH-26 Endofill Tubliseal Zinc oxide eugenol Apatite root canal sealer III gutta-percha and Cavizol	*Ex vivo*	Digital images	-	Endofill and ZnOE caused the greatest discolouration and Apatite root canal sealer III caused the least discolouration
**Ioannidis ** ***et al.*** ** [** 22 **]**	Roth 811 AH-26 GuttaFlow Epiphany SE	*Ex vivo*	UV–Vis spectrophotometer	80	Roth 811 sealer exhibited severe discoloration effects (*P*< 0.05)

In a spectrophotometric analysis by El Sayed and Etemadi [23], it was concluded that AH-Plus and Sultan (a ZOE-based sealer, Sultan Chemist Inc, Englewood, NJ, USA) may cause a progressive coronal discoloration effect over 10-17 days but Apexit Plus sealer showed the least coronal discoloration.

***Glass ionomer sealers***

Glass ionomer-based sealers have been advocated for use in obturation because of their sealing ability and adhesion to the root canal wall which causes monoblock obturation [24]. A disadvantage of these sealers is that they cannot be easily removed in case retreatment is required [25]. Ketac-Endo (3M ESPE, St. Paul, Minnesota) has minimal antimicrobial activity [26]. No study have evaluated the discoloration potential of glass ionomer-based sealers.

***Resin-based sealers***

Resin sealers have a long history of use. They provide adhesion to the root canal walls and are free of eugenol. These types of sealers fall into two major categories based on their resin content: epoxy resin-based [AH-26 (Dentsply, Tulsa Dental, Tulsa, OK, USA) and AH-Plus (Dentsply, Tulsa Dental, Tulsa, OK, USA)] and methacrylate resin-based sealers [EndoREZ (Ultradent Products Inc., UT, USA), MetaSeal is also marketed as Hybrid Bond Seal (Sun Medical Co. Ltd., Shiga, Japan) and RealSeal (SybronEndo, Orange, CA, USA)] [27, 28]. 

Almost all studies conducted on discoloration potential of different sealers have confirmed the high discoloration potential of this type of sealers. In *in vitro* settings AH-26 causes more tooth discoloration in comparison with a ZOE-based sealer (Dorifill) [17]. According to the results of another spectrophotometric study, the tooth discoloration after using sealer 26 was more than AH-Plus and a ZOE-based sealer (Dorifill) [16]. Lower discoloration potential of AH-Plus in comparison to resin sealer was confirmed in a study by Lenherr *et al.* [21] who showed minor tooth discoloration comparable to negative control group in teeth obturated with this sealer.

Considering the high discoloration potential of these sealers, their substitution with other sealing agents for treatment of anterior region or considering the level of root filling removal in the esthetic zone and elimination of sealer remnants from the pulp chamber walls must be considered. 

***Silicon-based sealers***

RoekoSeal (Coltène/Whaledent, Langenau, Germany) is a polydimethyl siloxane that present the unique feature of slight expansion on setting [29]. The material provides a working time of 15 min and sets after 25-30 min. Evidence suggests that this sealer is biocompatible; however, its setting time is inconsistent and may be delayed by final irrigation with sodium hypochlorite [30, 31]. GuttaFlow (Coltène/Whaledent Inc, Cuyahoga Falls, OH, USA) is a polyvinylsiloxane with finely milled gutta-percha particles added to RoekoSeal. The only study on discoloration potential of silicone-based sealers is conducted by Ioannidis *et al.* [22] under *in vitro* settings using spectrophotometric analysis. They demonstrated that teeth obturated with GuttaFlow had no clinically significant discoloration which was comparable to AH-26 and Epiphany. However, in their study, Roth 811 resulted in significant discoloration [22]. 

***Bioceramic sealers***

The main concept behind the development of bioceramic sealers, is the exploitation of their physical and biological properties such as bioactivity, biocompatibility and hard tissue conductivity [32]. Because of crown staining by the bismuth oxide component of these sealers, which may be rendered brown (in contact with NaOCl), gray (in contact with chlorhexidine) or even black (in contact with glutaraldehyde), this radiopacifier has now been replaced with other materials such as zirconia dioxide (zirconia) or tantalum oxide in some commercial formulations [5, 15]. Tricalcium silicate sealers are MTA Fillapex (Ângelus Indústria de Produtos Odontlógicos Ltda; Londrina, Paraná, Brazil), iRoot SP (Innovative BioCeramix Inc., Vancouver, Canada; aka Endosequence BC sealer; Brasseler USA), Endo CPM Sealer (EGEO SRL, Buenos Aires, Argentina) and MTA Plus (Avalon Biomed, Bradenton, Florida), Sankin Apatite Root Canal Sealer (SARCS) (Sankin kogyo, Tokyo, Japan). According to loannidis *et al.* [15] MTA Filapex induced minimal coronal discoloration in comparison with Roth 811.

In an *in vitro* computer analysis of crown discoloration by Partovi *et al.* [5] it was shown that after nine months, AH-26, Endofill, TubliSeal, ZOE and Sankin Apatite Root Canal Sealer (SARCS) type III (a bioceramic sealer with individual apatite-like crystallites; Sankin kogyo, Tokyo, Japan) caused some degrees of tooth discoloration, which increased with time. Endofill and ZNO caused the greatest discoloration and SARCS caused the least discoloration after 9 months. The most discoloration during the test periods occurred in the cervical third of the crown [5]

## Conclusion

There is only scarce or no evidence available on the staining potential of endodontic sealers currently available on the market. Therefore, endodontic therapy should not only focus solely on biological and functional aspects, but also must take aesthetic considerations into account. To reduce the risk of tooth discoloration, all endodontic sealers should be applied carefully in areas of aesthetic concern. 
